# Supplementary material for: Eliminating effects of particle adsorption to the air/water interface in single-particle cryo-electron microscopy: Bacterial RNA polymerase and CHAPSO
Source: J Struct Biol X. 2019 Feb 14;1:100005. doi: 10.1016/j.yjsbx.2019.100005 (PMC7153306; doi:10.1016/j.yjsbx.2019.100005)
Supplement: Supplementary data 5 [file mmc5.docx]

**Supplemental Table 1**. Solution conditions for 6S-Eσ^70^ cryo-EM analyses.

| Abbreviation | Solution components | [RNAP] (μM) |
| --- | --- | --- |
| KGlu | 10 mM Tris-HCl, pH 8.0, 150 mM K-glutamate, 1 mM MgCl_2_, 2 mM DTT | 0.9 |
| KCl | 10 mM Tris-HCl, pH 8.0, 150 mM KCl, 1 mM MgCl_2_,  2 mM DTT | 0.9 |
| KGlu-NP40S | 10 mM Tris-HCl, pH 8.0, 150 mM K-glutamate, 1 mM MgCl_2_, 2 mM DTT, 0.06 mM Nonidet P40 substitute (NP40S) | 6 |
| KCl-CHAPSO | 10 mM Tris-HCl, pH 8.0, 150 mM KCl, 1 mM MgCl_2_,  2 mM DTT, 8 mM CHAPSO | 9 |

**Supplemental Table 2**. Screening of cryo-EM solution conditions for 6S-Eσ^70^.

| **Solution Conditions** | **EMSA^a^** | **Negative Stain EM** | **Cryo-EM** |
| --- | --- | --- | --- |
| 10mM Tris-HCl pH 8.0, 150 mM KGlu, 1 mM MgCl_2_, 2 mM DTT | + | + | + |
| 10 mM Tris-HCl pH 8.0, 150 mM KGlu, 1 mM MgCl_2_, 2 mM DTT, 0.15 mM DDM | + | - | - |
| 10 mM Tris-HCl pH 8.0, 150 mM KGlu, 1 mM MgCl_2_, 2 mM DTT, 0.18 mM Triton™ X-100 | - | - | - |
| 10 mM Tris-HCl pH 8.0, 150 mM KGlu, 1 mM MgCl_2_, 2 mM DTT, 0.04mM TWEEN® 20 | - | - | - |
| 10 mM Tris-HCl pH 8.0, 150 mM KGlu, 1 mM MgCl_2_, 2 mM DTT, 0.06 mM NP40-Subtitute | + | + | + |
| 10 mM Tris-HCl pH8.0, 150 mM KGlu, 1 mM MgCl_2_, 2 mM DTT, 8 mM CHAPSO | + | - | - |
| 10 mM Tris-HCl pH8.0, 150 mM KCl, 1 mM MgCl_2_, 2 mM DTT | + | + | + |
| 10 mM Tris-HCl pH8.0, 150 mM KCl, 1mM MgCl_2_, 2 mM DTT, 8 mM CHAPSO | + | + | + |

^a^ electrophoretic mobility shift assay

^†^ + indicates experiments that show that the 6S-Eσ^70^ complex is stable in a given buffer condition, whereas – indicates experiments that show destabilization of the 6S-Eσ^70^ complex in a given buffer condition. Green highlights conditions used for single particle cryo-EM.

**Supplemental Table 3.** Data collection and reconstruction information for single particle cryo-EM datasets.

|  | **KGlu** | **KCl** | **KGlu NP40S** | **KCl CHAPSO** | **TEC** | **TEC + 4mM CHAPSO** | **TEC + 8mM CHAPSO** |
| --- | --- | --- | --- | --- | --- | --- | --- |
| **Sample** |  |  |  |  |  |  |  |
| Buffer | 10mM Tris-HCl pH 8.0, 150mM KGlu, 1mM MgCl_2_, 2mM DTT | 10mM Tris-HCl pH8.0, 150mM KCl, 1mM MgCl_2_, 2mM DTT | 10mM Tris-HCl pH8.0, 150mM KGlu, 1mM MgCl_2_, 2mM DTT, 0.06mM NP40-Subtitute | 10mM Tris-HCl pH8.0, 150mM KCl, 1mM MgCl_2_, 2mM DTT, 8mM CHAPSO | 20mM Tris pH8.0, 150mM KCl, 5 mM MgCl_2_, 5mM DTT | 20mM Tris pH8.0, 150mM KCl, 5 mM MgCl_2_, 5mM DTT, 4mM CHAPSO | 20mM Tris pH8.0, 150mM KCl, 5 mM MgCl_2_, 5mM DTT, 8mM CHAPSO |
| Grid type | Quantifoil | Quantifoil | Quantifoil | Quantifoil | C-Flat | C-Flat | C-Flat |
| **Data Collection** |  |  |  |  |  |  |  |
| Microscope | Tecnai G2 Polara | Tecnai G2 Polara | Tecnai G2 Polara | Tecnai G2 Polara | Titan Krios | Talos Arctica | Ttitan Krios |
| Voltage (keV) | 300 | 300 | 300 | 300 | 300 | 200 | 300 |
| Nominal Magnification | 31000x | 31000x | 31000x | 31000x | 22500x | 28000x | 22500x |
| Number of frames/movie (no.) | 30 | 30 | 30 | 30 | 50 | 50 | 50 |
| Exposure rate  (e-/pixel/sec) | 8 | 8 | 8 | 8 | 10 | 10 | 10 |
| Total exposure time (sec) | 6 | 6 | 6 | 6 | 15 | 15 | 15 |
| Electron dose  (e-/Å^2^) | 31.7 | 31.7 | 31.7 | 31.7 | 88.6 | 66.7 | 88.6 |
| Detector | K2 Summit | K2 Summit | K2 Summit | K2 Summit | K2 Summit | K2 Summit | K2 Summit |
| Recording mode | Counting | Counting | Counting | Counting | Super-resolution | Super-resolution | Super-resolution |
| Pixel size (Å) | 1.23 | 1.23 | 1.23 | 1.23 | 1.3 | 1.5 | 1.3 |
| Defocus Range (μm) | -1.3 to -1.7 | -1.4 to -2.2 | -1.5 to -2.3 | -1.4 to -2.3 | -1.5 to -3.5 | -0.8 to -2.4 | -2.5 to -5.0 |
| **Reconstruction** |  |  |  |  |  |  |  |
| Number of micrographs (no.) | 215 | 62 | 147 | 290 | 500 | 558 | 491 |
| Number of particles picked (no.) | 40542 | 26114 | 33987 | 39002 | 68994 | 134546 | 65659 |
| Symmetry Imposed | C1 | C1 | C1 | C1 | C1 | C1 | C1 |
| Number of particles after 2D (N=50) (no.) | 24061 | 20487 | 29153 | 12540 | 23960 | 41399 | 28130 |
| Number of particles after 3D (N=3) (no.) | 12950 | 9332 | 16695 | 5978 | 11410 | 29513 | 12066 |

**Supplemental Table 4.** Data collection and reconstruction information for cryo-ET datasets.

|  | **KGlu** | **KCl** | **KGlu NP40S** | **KCl CHAPSO** |
| --- | --- | --- | --- | --- |
| **Data collection** |  |  |  |  |
| Buffer | 10mM Tris-HCl pH 8.0, 150mM KGlu, 1mM MgCl_2_, 2mM DTT | 10mM Tris-HCl pH8.0, 150mM KCl, 1mM MgCl_2_, 2mM DTT | 10mM Tris-HCl pH8.0, 150mM KGlu, 1mM MgCl_2_, 2mM DTT, 0.06mM NP40-Subtitute | 10mM Tris-HCl pH8.0, 150mM KCl, 1mM MgCl_2_, 2mM DTT, 8mM CHAPSO |
| Grid type | Quantifoil | Quantifoil | Quantifoil | Quantifoil |
| Microscope | Titan Krios | Titan Krios | Titan Krios | Titan Krios |
| Camera | K2 Summit | K2 Summit | K2 Summit | K2 Summit |
| Magnification | 18,000x | 18,000x | 18,000x | 18,000x |
| Recording mode | Counting | Counting | Counting | Counting |
| Voltage (keV) | 300 | 300 | 300 | 300 |
| Exposure navigation | Stage Position | Stage Position | Stage Position | Stage Position |
| Total dose (e-/Å^2^) | 118 | 118 | 118 | 118 |
| Exposure rate (e-/pixel/sec) | 8 | 8 | 8 | 8 |
| Pixel size (Å) | 1.33 | 1.33 | 1.33 | 1.33 |
| Number of frames/movie (no.) | 8-11 | 8-11 | 8-11 | 8-11 |
| Nominal defocus (µm) | 5 | 5 | 5 | 5 |
| Tilt range and increment (˚) | -45:45:3 | -45:45:3 | -45:45:3 | -45:45:3 |
| Tilt-series collected | 3 | 3 | 4 | 3 |
| Particles in each tomogram shown in Figure 2 | 266 | 307 | 894 | 107 |
